# The Association of Social Media Use and Eating Behaviour of Belgian Adolescent Girls Diagnosed with Anorexia Nervosa—A Qualitative Approach

**DOI:** 10.3390/children11070822

**Published:** 2024-07-04

**Authors:** Nathalie Praet, Jeff Stevens, Kristina Casteels, Jaan Toelen

**Affiliations:** 1 Faculty of Medicine, KU Leuven, 3000 Leuven, Belgium; nathalie.praet@student.kuleuven.be (N.P.); jeff.stevens@student.kuleuven.be (J.S.); 2 Department of Paediatrics, University Hospitals Leuven, 3000 Leuven, Belgium; kristina.casteels@uzleuven.be; 3 Department of Development and Regeneration, KU Leuven, 3000 Leuven, Belgium; 4 Child and Youth Institute, KU Leuven, 3000 Leuven, Belgium

**Keywords:** selective content, biased interpretation, behavioural adaptation, recovery process, body dissatisfaction, comparison

## Abstract

Background: Social media have become integral in adolescents’ lives, presenting both opportunities and risks, especially concerning psychiatric issues like eating disorders, prevalent in this vulnerable age group. Methods: This qualitative study employed semi-structured interviews with seven adolescent girls (aged 15–17) diagnosed with eating disorders. Interviews covered seven predefined topics, recorded and transcribed for thematic analysis. Results: Participants identified four key themes: exposure to selective content, biased interpretation, behavioural adaptation, and evolving perspectives during recovery. They highlighted social media’s role in exacerbating body dissatisfaction and altering behaviours related to eating disorders. Conclusions: This research underscores the critical need for awareness and guidance in adolescents’ social media use to mitigate negative impacts, emphasizing the potential link between exposure to specific content and cognitive-behavioural changes in those with eating disorders. Further investigation is warranted to deepen our comprehension of these dynamics.

## 1. Introduction

A substantial majority (97%) of adolescents in industrialized nations engage with social media regularly, signifying its integral role in their daily lives and broader societal dynamics [1]. Over the last few years, traditional media have been increasingly replaced by social media, which offer both active and passive use. These platforms are continuously accessible and provide a vast array of information, entertainment, social interaction, and marketing opportunities [2]. Consequently, social media significantly influence adolescents’ social and emotional development [1].

Children and adolescents are engaging with social media at younger ages as mobile device ownership becomes more common, leading to the formation of their social media portfolios at a considerably younger age [2]. Gender differences in social media use are evident: girls predominantly use platforms like Instagram and Tumblr, whereas boys prefer Facebook. Girls tend to maintain private accounts and frequently post personal content, while boys are more likely to have public accounts and share memes and possessions. These gender-based differences in social media use are associated with varied impacts on boys and girls [3].

The increased use of social media among adolescents exposes them to potential risks, including pornography, violence, and cyberbullying, affecting approximately 15% of this population. These exposures have been linked to depression, anxiety, and lowered self-esteem. Conversely, positive use of social media can enhance mental well-being by strengthening peer relationships, social skills, and support networks, thereby reducing stress [4]. Adolescents often perceive these negative consequences as more impactful on others than on themselves, a phenomenon known as the ‘personal fable’, influenced by their developmental stage [1].

The prevalence of eating disorders has increased significantly, with a 12.5% rise since 1990, resulting in a lifetime prevalence of 13% [5]. Anorexia nervosa (AN), bulimia nervosa (BN), and binge eating disorder (BED) are now among the most diagnosed eating disorders, with AN being particularly prevalent in clinical settings. Adolescent girls are the most susceptible to eating disorders, with a lifetime prevalence of 4% for developing AN [6,7,8]. AN is characterized by significant underweight, fear of gaining weight, and a disturbed self-image [6].

Previous research suggests a link between social media use and the development of eating disorders. Network analysis of studies on body image reveals that exposure to ‘idealized body appearance’ images on social media is a significant factor [9]. ‘Fitspiration’, a social media trend promoting a healthy lifestyle, has varied effects. Studies show that young women exposed to fitspiration on Instagram often internalize the ‘thin ideal’, leading to increased body dissatisfaction [3,10,11,12]. Similarly, TikTok users exposed to such content experience higher body dissatisfaction and reduced self-esteem due to frequent comparisons [13,14,15]. Body dissatisfaction is a well-established precursor to eating disorders [16,17,18,19]. This suggests that social media may contribute to eating disorders by fostering body dissatisfaction.

Despite growing awareness of social media’s impact on mental health, most studies have focused on adolescents or adults without pre-existing mental illnesses. There is a lack of research on the influence of social media on individuals diagnosed with eating disorders. This study aims to explore through qualitative semi-structured interviews whether adolescents with eating disorders perceive social media as influencing their illness.

## 2. Materials and Methods

### 2.1. Research Design

This study employed a qualitative approach utilizing semi-structured interviews to understand participants’ perspectives comprehensively. Semi-structured interviews facilitate the flexibility for detailed exploration of specific viewpoints, allowing for a nuanced understanding of individual experiences. Given the emphasis on personal experiences, a qualitative methodology was chosen over a quantitative survey.

### 2.2. Design and Validation of the Interview Guide

The development of the interview guide was based on an extensive review of existing literature on eating disorders and their correlation with social media. Multiple medical databases were queried to gather publications concerning the impact of social media on adolescents, biological, psychological, and social changes during adolescence, comprehensive insights into eating disorders, and the current understanding of the interplay between eating disorders and social media. This literature review aimed to establish a solid theoretical framework for the research. Drawing from this theoretical foundation, a structured interview guide was formulated, comprising approximately thirty questions categorized into seven main domains: general social media use, positive and negative outcomes of social media engagement, exposure to specific content on social media platforms, social interactions facilitated by social media, influence of social media on eating behaviours and eating disorders, and social media platforms dedicated to eating disorders. The interview guide followed a gradual approach, initially exploring participants’ general social media habits before delving into the connection between their eating disorders and social media. Open-ended questions allowed participants to offer personal interpretations and insights.

### 2.3. Study Settings and Participants

Participants were recruited from the UZ Leuven paediatrics department by staff members. Inclusion criteria encompassed female adolescents aged 12 to 17 diagnosed with anorexia nervosa (DSM V, restrictive type). We restricted our sample to female participants to limit divergence. The time to diagnosis of AN was not utilized as inclusion or exclusion criteria in this study. The paediatricians assessed the candidates’ eligibility for participation. Individuals for whom participation was deemed excessively challenging (due to clinical or psychological concerns) were excluded.

### 2.4. Data Collection/Interview Process

Following invitation from clinical staff, potential participants received detailed information about the research via a letter to enable informed decision making. Researchers contacted interested candidates, discussing the informed consent process thoroughly before each interview. Participation was voluntary and non-binding, ensuring the freedom to withdraw at any point without repercussions. Written informed consent was obtained from the parents due to participants’ minor status, while participants provided verbal assent. Interviews were conducted via live video calls on Teams, with audio recordings obtained with participant consent for transcription purposes. Recordings were stored anonymously and deleted after transcription. Each interview lasted approximately 90 min, followed by an opportunity for participants to address any queries or share reflections on the process. Researchers remained available for further questions or support.

### 2.5. Data Analysis

Manual transcription of interviews was followed by coding procedures. “Open coding” involved summarization and concept construction through labelling by researchers, with regular meetings to reconcile discrepancies and establish common labels. Subsequently, a “preliminary coding framework” was developed based on these summaries. “Axial coding” introduced overarching categories applicable across all interviews, while “selective coding” finalized the inclusion or exclusion of data.

### 2.6. Ethical Considerations

The research adhered to the ethical principles endorsed by the Research Ethics Committee UZ/KU Leuven (MP024076), aligning with ICH-GCP standards, Helsinki Declaration, and relevant laws. Informed consent highlighted voluntary and anonymous participation, with participants being informed about audio recordings and their eventual deletion. No identifiable information was collected, and ethical considerations included offering access to psychologists if needed, with researchers available for queries.

## 3. Results

### 3.1. Demographics

Seven Caucasian adolescent girls aged 15 to 17, diagnosed with restrictive anorexia nervosa, participated in the study. The average age of the participants was 15 years and 10 months, with an average eating disorder duration of 1 year and 6 months at the time of the interview. Initial BMI at onset ranged from 11.6 to 17.6, with a mean of 15.1. At the time of the interviews, BMI ranged from 15.9 to 22, with an average of 18.2. During the last three interviews, no new themes or subthemes were identified, suggesting answer saturation.

### 3.2. Overview of Themes and Subthemes

**Figure 1:**
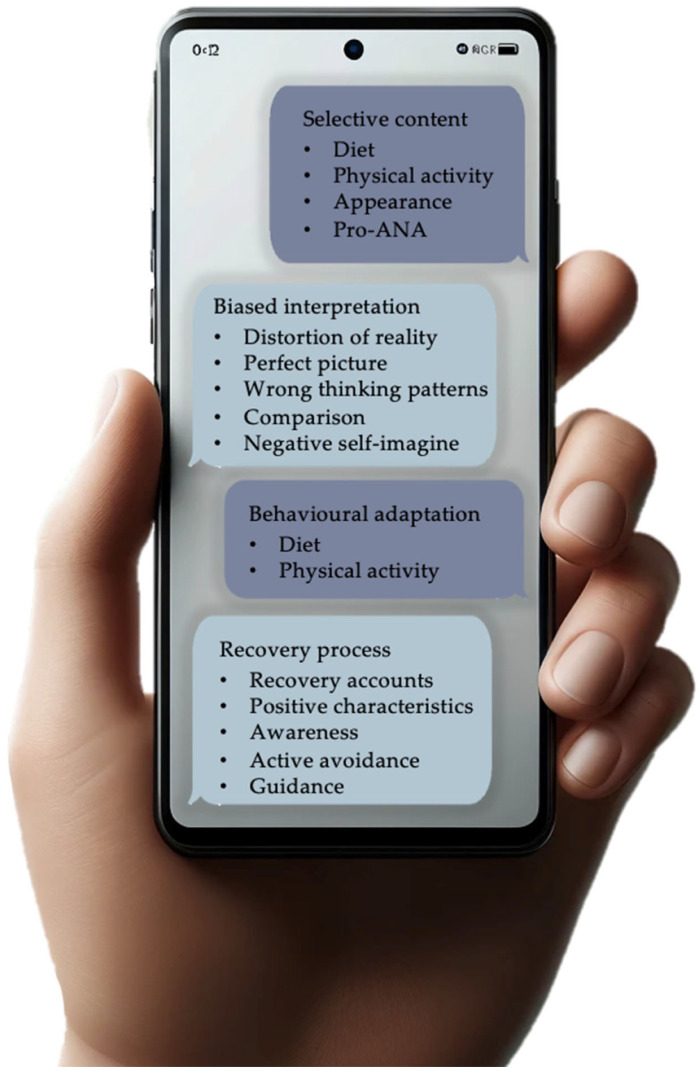
Overview of the main themes and subthemes influenced by social media.

The coding process identified four major themes: selective content, biased interpretation, behavioural adaptation, and recovery process. These themes were further divided into specific subthemes (see Figure 1).

### 3.3. Selective Content

Adolescents with eating disorders reported exposure to triggering content on social media. Some participants noted that they encountered such content even before their diagnosis, believing it may have contributed to the disorder’s development. Pro-ANA materials were described as addictive, making active avoidance challenging. Eventually, they actively sought this content due to an intrinsic urge, leading to increased exposure as algorithms aligned with their interests.

*Participant 3: “I think it is a kind of addiction after all, because I keep looking for the positive but then the negative always comes back.”*

*Participant 1: “Back then the content was all about eating, eating, eating and how meals were made, and how many calories it contained. If I didn’t know the caloric content, I also pretended not to be interested in the hope that I would get other content including the calories.”*

Participants encountered three types of eating disorder-related content: food, physical activity, and physical appearance. First, they were exposed to online content related to diet and food intake. These online materials promoted a restrictive and selective diet. A substantial portion of the content to which they were exposed pertained to caloric content of various food items, and they received guidance to abstain from food with high caloric values. Consequently, exposure to “What I Eat in A Day” content engendered an excessive preoccupation with their food intake quantities. In addition to the avoidance of specific food items, recommendations for healthier food alternatives were provided.

*Participant 3: “Very often counting calories was encouraged and suggestions were made like: ‘these are healthier snacks, or these foods are not okay to consume.’ Also, ‘What I Eat in A Day’-videos but it was very little and hyper healthy.”*

*Participant 6: “I primarily monitored caloric intake myself, so I would search for this kind of information on the internet and occasionally encounter it on social media, such as individuals substituting bread with bell peppers, for example.”*

The second type of content the participants preferred was related to physical activity, which provided tips for rapid weight loss. The combination of content related to maximal caloric expenditure during exercise and daily life, along with content related to reduced caloric intake, resulted in a negative energy balance.

*Participant 7: “Sports that burn the most in a shorter time and so on. Climbing stairs and jumping rope and so on. And never sitting still with my legs, that I always have to move them when I sit.”*

The final category of preferred content pertains to physical appearance. Participants exhibited a marked preference for content that idolized the slender physiques of models and influencers. This content contributed to the formation of an ‘ideal body image’ to which the participants aspired.

*Participant 3: “Very often images of someone who is underweight or who is more like the ideal image and if you have an eating disorder you would look like that.”*

### 3.4. Biased Interpretation

Participants showed a biased interpretation of online content, focusing on diet, physical activity, and appearance, even when this content was unrelated to eating disorders. This led to a distorted reality, often overlooking digital image manipulation. During recovery, their perception of reality shifted, as will be discussed later.

*Participant 7 on selective focus: “Yes, I do look more at people’s appearance and body and also, yes, still nutrition and so on and in terms of exercise. What people do. Before my eating disorder, I used to look mainly at creative things and cheerful things.”*

*Participant 1 on reflection of reality: “I see someone thin, and I want to be like that, or I want to be thinner. Social media was just the perfect image for me, and I had to meet those standards, and I saw the reality in that photo, and you don’t immediately think: ‘that could also be photoshopped’”.*

The distorted reflection led to an incorrect thought pattern throughout the course of the eating disorder, resulting in a mindset that was equally fixated on the eating disorder and social approval. As previously indicated in the earlier quotations, one of the key factors is the comparison that the participants drew between the continuous triggering content on social media and themselves. The comparison applied to all previously mentioned content (food, physical activity and physical appearance), which resulted in negative emotional states and sometimes a preoccupation with certain body parts. This, in turn, gave rise to a negative spiral, resulting in self-disappointment, feelings of loneliness due to the emergence of a sense ‘not to be good enough’, and the development of shame and jealousy.

*Participant 1: “Advertisements from people who would lose weight and say: ‘now I’m much happier’, then you want to be like that too, so you want to comply, and you want to lose weight to be happier too”.*

*Participant 3: “Yes, feeling sad, becoming very insecure about yourself. I also think that there is a certain fear of not fitting in or not being like the people on social media, so will I be accepted by society?”*

### 3.5. Behavioural Adaptation

Negative self-image and false thinking patterns are suggested to be the basis for behavioural changes, including adopting low-calorie recipes, avoiding specific foods or restricting food intake to certain times of the day. Additionally, engaging in extreme exercise and/or physical activity was observed, accompanied by a shift in actively seeking certain content, as stated above.

*Participant 1: “For example, articles that advise you to eat more slowly and chew well. So yeah, it can apply to a lot of things: what you eat, how you eat, how much you eat, it can apply to everything.”*

*Participant 2: “Oh, look at them, they eat at that time, and they have their last meal at that time, and then I start thinking: ‘I shouldn’t eat before that hour, and I definitely shouldn’t eat after that hour. I can only drink this and I definitely can’t drink that and can only eat this’.”*

*Participant 4: “When I was in the midst of my eating disorder, it was mainly about things related to food, you know. A lot of ‘What I Eat in A Day’ stuff. And then, I was sort of comparing myself, and I was constantly looking at it to make sure I ate less.”*

### 3.6. Recovery Process

Throughout the recovery process, a notable shift was observed in how participants interpreted online content, resulting in a different understanding and impact of digital materials. Initially, identifying triggering content was essential, and recognizing the impact of these comparisons on their emotional state was crucial. By altering their interpretation, participants gained insight into the distorted portrayals on social media and their adverse effects on recovery. This shift often led to actively avoiding specific social media content. Unlike the early stages of the illness, where there was a selective focus on eating disorder-related content, adopting an avoidant approach proved vital during recovery.

*Participant 2 about awareness: “A lot of my friends use Facebook and so on, but I’ve already said, for example, that I’d rather not because I’m afraid of social media, because I know it can have an impact anyway”.*

*Participant 5 about active avoidance: “Yeah, as I mentioned earlier, those negative accounts, I just block them or something.”*

Guidance from parents and caregivers was also essential in transforming the interpretation of online content and reducing negative comparisons. Counselling and supervision of social media use by parents and caregivers could assist adolescents in developing awareness and actively avoiding negative aspects of their social media use. Several participants emphasised that the role of this guidance should not be underestimated, as it expedites the transformation of the interpretation of online content and triggers, ultimately leading to an end of the continuous negative comparisons that were being made.

*Participant 1: “You can’t yet be aware that something is triggering, and if you’re already aware of that and then dare to indicate that I don’t really like this kind of content, I want to have something else on my social media, then I think you have to find your way in that, but that will come when you have guidance. I think it’s hard to beat an eating disorder alone, the path that must be walked together.”*

In addition to the potential for positive evolution through social media use, various types of content on these platforms were observed to contribute to this transformation. This includes content related to eating disorders, such as recovery testimonials, as well as other content categories. Recovery accounts facilitated positive evolution in participants by presenting content that highlighted achievable outcomes if they overcame their eating disorder. This content helped in breaking stereotypes or taboos, alleviated feelings of loneliness by demonstrating shared experiences, promoted body positivity and self-love, and provided challenges related to eating. These elements seemed to trigger positive emotions in participants, such as recognition and motivation, and provided tools to challenge distorted thinking patterns, fostering a hopeful future perspective. However, it is important to note that these accounts could also serve as triggers when participants were in a negative emotional state, potentially leading them to perceive recovery as unattainable and inducing a sense of despair.

*Participant 2: “That you are not allowed to feel bad, and that food is actually something that you need to live and to do things that you like to do. Trying to promote that kind of stuff, think about it carefully and not see it too negatively and that you are also allowed to enjoy something lesser, that it may not always be very healthy.”*

*Participant 5: “These are mostly people who have experienced an eating disorder themselves. They share their experiences, detailing their journey in recovery and how it has positively influenced their choice to pursue and continue the path of healing.”*

Contact with peers, distraction, and inspiration from social media also contributed positively, helping to limit feelings of isolation and providing them with a degree of connection. Furthermore, social media could serve as a source of diversion, with it being perceived as a form of relaxation and creative inspiration for specific projects, fashion, or personal interests.

*Participant 4: “I think that at times, I have certainly derived positive things from social media, such as friends who were there for me and sent me uplifting messages.”*

*Participant 5: “Personally, I also draw a lot of inspiration, yeah, for creative pursuits. Or from fashion, outfit inspiration, and such.”*

## 4. Discussion

This study explored how adolescents with anorexia nervosa perceive social media content. Previous research indicates that social media influences disordered eating patterns, with exposure to “ideal beauty standards” leading to body dissatisfaction and unhealthy eating behaviours [20,21]. Once confined to magazines [22,23,24], this exposure has now increased with social media, worsening body dissatisfaction and problematic eating [3,25,26]. Recent studies suggest a causal link between social media and disordered eating. Participants who abstained from social media for a week showed fewer eating disorder symptoms compared to a control group [27].

Despite the growing evidence linking social media with eating disorders, research on its impact on adolescents already diagnosed with an eating disorder is limited. Our qualitative study identified four main themes regarding the influence of social media on the course of eating disorders: selective content (diet, physical activity, appearance, and pro-ANA), biased interpretation (distortion of reality, perfect picture, wrong thinking patterns, comparison, negative self-image), behavioural adaptation (diet and physical activity), and the recovery process (awareness, active avoidance, guidance, positive characteristics, and recovery accounts).

The first main theme concerns the content of social media and its link to eating disorders. Our data indicate that social media content related to physical appearance significantly influences both the initiation and perpetuation of eating disorders. This is consistent with other studies showing that adolescents compare themselves to unrealistic portrayals of bodies, leading to body dissatisfaction and unhealthy eating behaviours [28,29]. This trend, often termed “thinspiration” or “fitspiration”, was frequently encountered by our study participants [30,31,32].

Our data revealed that adolescents in the early stages of eating disorders actively seek content related to food intake and fitspiration. Videos like “What I Eat in A Day” serve as consumption benchmarks [33], potentially distorting thought patterns regarding daily food intake even among healthy adolescents [34]. Moreover, pro-ANA (pro-anorexia) content was identified as triggering by our participants. Previous studies already documented its adverse impact on the quality of life and its potential to prolong eating disorders [35]. Participants noted that seeking food and pro-ANA content led to more exposure due to algorithmic recommendations [36].

The second major theme involved the distorted interpretation of social media content. Previous studies have shown higher rates of eating disorder-related behaviours and self-comparison in individuals who spend more time on social media [37,38]. This comparative behaviour is influenced by peers [39] and influencers who promote an idealized body image, negatively impacting mood and body dissatisfaction [40]. Our data highlight the central role of comparison in perpetuating negative emotions, with photo manipulation tools like Photoshop exacerbating body dissatisfaction [41,42].

The third theme, behavioural adaptation, specifically concerns changes in food intake and physical activity. Prior studies have identified both direct and indirect links between social media exposure and eating behaviours among healthy adolescents and young adults [43]. Exposure to fitspiration and thinspiration was associated with disordered eating behaviours, such as purging and restrictive dieting, in female university students [27]. Our findings align with these observations, with participants describing shifts towards more selective and restrictive diets influenced by online examples. A recent review article corroborated this relationship, noting the association between exposure to food-related content on social media, body image problems and disturbed eating behaviour [44]. A randomized controlled trial of adolescent girls found that more social media use led to greater influence on distorted food intake thoughts and behaviours in adolescent girls [3].

Participants reported that social media examples of physical activity encouraged extreme and unhealthy habits. This aligns with research showing postpartum women exposed to appearance-focused social media exhibited more distorted eating and unhealthy physical activity desires [45]. Conversely, social media can also promote healthy habits as demonstrated by a cross-sectional analysis where one-third of adolescents with obesity increased their physical activity through social media [40]. Given social media’s dual impact on adolescents’ physical activity behaviours, it seems to be crucial to provide guidance and education on appropriate social media use.

The final theme concerns social media’s role in the recovery process. When used appropriately, social media can support recovery by facilitating interaction with online recovery communities. An online qualitative survey of adults engaged with recovery accounts on Instagram highlighted both positive and negative aspects of social media during recovery, similar to our findings [46]. Contact with peers on recovery accounts fosters a sense of community and understanding, with recovery journeys serving as motivation and inspiration.

We identified three primary factors influencing recovery: awareness, active avoidance, and guidance. Participants’ growing awareness of social media’s impact led to more mindful use, often resulting in the active avoidance of certain media. This selective use, documented in other studies, correlates with decreased eating disorder-related cognition and behaviours [3,44]. Furthermore, educational interventions on social media literacy have been shown to reduce risk factors for developing eating disorders, highlighting the potential of guidance in facilitating awareness and active avoidance [46]. However, social media can also expose users to certain triggers during recovery. One study highlighted various negative consequences of using recovery accounts, such as the underrepresentation of different body types, content focused on appearance, and misinformation [47], aspects not observed in our study.

Our study has several limitations. During the recruitment process, it became evident that many adolescents diagnosed with an eating disorder were reluctant to participate due to the difficulty of discussing the subject. However, after five interviews, no new themes emerged, suggesting answer saturation. Therefore, we consider the number of participants to be sufficient. Given that this study employs a qualitative approach with in-depth interviews in a homogenous group, the primary focus is on understanding the “how” and exploring the “various relationships between the investigated categories” rather than proving a specific hypothesis [48,49]. Malterud et al. (2016) introduced the concept of information power to assess sample size. Given our narrow study aim, highly specific participant selection and the desire for in-depth analysis, a smaller sample size is sufficient [50].

The qualitative nature of our study makes it susceptible to various biases that could influence our findings and interpretations. To address selection bias, we carefully defined inclusion and exclusion criteria and specified the study population beforehand. Notably, the average duration of the eating disorder among participants was 18 months, indicating they were in a more advanced stage of recovery with significant experience of social media’s influence.

During interviews, the primary researchers used a guiding rather than a directive approach to prevent interviewer bias. The interview guide started with broad, general questions before moving to more detailed and specific topics, minimizing the influence of question sequencing. Additionally, the interviews were conducted by the primary researchers rather than the treating physicians, allowing participants to speak freely and thus minimizing response bias. The use of semi-structured interviews with specific main questions may have led to the exclusion of certain topics, potentially missing essential triggers. Our focus was on the eating disorder process itself, rather than solely on the recovery process, which may explain the fewer themes identified compared to other studies. Additionally, the interview structure allowed for sub-questions based on respondents’ answers, leading to a more extensive exploration of some topics. This may have created the impression that these topics played a more significant role in maintaining the eating disorder or influencing the recovery process.

## 5. Conclusions

In conclusion, this study examined the impact of social media on adolescents with anorexia nervosa through semi-structured interviews. Four key themes emerged, illustrating the complex relationship between social media and eating disorders:

- Content focused on eating disorders: Social media content related to physical appearance promotes unrealistic body ideals, leading to body dissatisfaction and unhealthy eating behaviours.
- Biased interpretation: Comparison and distorted perceptions, often influenced by social media influencers and photo manipulation, contribute to negative emotions and persistent body dissatisfaction.
- Behavioural adaptation: Exposure to social media content alters food intake and physical activity patterns.
- Recovery process: Awareness and active avoidance of social media can aid recovery, though guidance is necessary to harness social media’s positive aspects.

The study underscores the importance of continued research to validate and understand the diverse impacts of social media on eating disorders, highlighting both potential triggers and beneficial aspects.

## Data Availability

Data available on request due to restrictions regarding privacy and ethical concerns. The data presented in this study are available on request from the corresponding author.
